# A folate receptor-targeted lipoplex delivering interleukin-15 gene for colon cancer immunotherapy

**DOI:** 10.18632/oncotarget.10537

**Published:** 2016-07-11

**Authors:** Xiao Liang, Min Luo, Xia-Wei Wei, Cui-Cui Ma, Yu-Han Yang, Bin Shao, Yan-Tong Liu, Ting Liu, Jun Ren, Li Liu, Zhi-Yao He, Yu-Quan Wei

**Affiliations:** ^1^ State Key Laboratory of Biotherapy and Cancer Center, West China Hospital, Sichuan University, Chengdu, Sichuan 610041, China

**Keywords:** interleukin-15, folate receptor α, colon cancer, immunotherapy, gene therapy

## Abstract

Interleukin-15 has been implicated as a promising cytokine for cancer immunotherapy, while folate receptor α (FRα) has been shown to be a potentially useful target for colon cancer therapy. Herein, we developed F-PLP/pIL15, a FRα-targeted lipoplex loading recombinant interleukin-15 plasmid (pIL15) and studied its antitumor effects *in vivo* using a CT26 colon cancer mouse model. Compared with control (normal saline) treatment, F-PLP/pIL15 significantly suppressed tumor growth in regard to tumor weight (*P* < 0.001) and reduced tumor nodule formation (*P* < 0.001). Moreover, when compared to other lipoplex-treated mice, F-PLP/pIL15-treated mice showed higher levels of IL15 secreted in the serum (*P* < 0.001) and ascites (*P* < 0.01). These results suggested that the targeted delivery of IL15 gene might be associated with its *in vivo* antitumor effects, which include inducing tumor cell apoptosis, inhibiting tumor proliferation and promoting the activation of immune cells such as T cells and natural killer cells. Furthermore, hematoxylin and eosin staining of vital organs following F-PLP/pIL15 treatment showed no detectable toxicity, thus indicating that intraperitoneal administration may be a viable route of delivery. Overall, these results suggest that F-PLP/pIL15 may serve as a potential targeting preparation for colon cancer therapy.

## INTRODUCTION

As a common malignant tumor in the gastrointestinal tract, colon cancer has the highest incidence of cancers affecting the digestive system [1, 2]. Although many therapeutic modalities including surgery, resection and chemotherapy have been established, the treatment of colon cancer is still far from ideal [2, 3]. Therefore, the development of novel, less toxic therapeutic agents is imperative to reducing the high mortality and morbidity rates associated with colon cancer [4–6].

Gene therapy is a treatment approach that involves the delivery of DNA, RNA, small interfering RNAs or antisense oligonucleotides [7–9]. Thus far, most of the clinical trials in gene therapy have been targeting cancer treatment (64.4% of all gene therapy trials) [10, 11] with remarkable efficacies noted both *in vitro* and *in vivo* [12–16]. These strategies typically include tumor cell apoptosis induction, tumor suppressor gene reintroduction, immunomodulation, oncogene inactivation and sensitivity gene introduction [12, 17]. In recent years, an increased recognition of a link between inflammation and the development of cancer has led to the development of cancer immunotherapies, which are designed to stimulate the immune system into rejecting and destroying tumors [18, 19]. Of these immunomodulators, interleukin-15 (IL15), a potent pro-inflammatory cytokine, has emerged as a candidate immunomodulator for the treatment of colon cancer [20–22].

IL15, which has a similar structure to interleukin-2 (IL2), is a member of the four α-helix bundle family of cytokines and was first identified in the supernatant of the monkey epithelial cell line CV-1/EBNA [23]. Furthermore, IL15 plays an important role in various diseases, including tumor modulation [24]. While IL15 functions in the activation of immune cells, such as B cells, DC cells, natural killer (NK) cells and T cells, its antitumor effects are executed by enhancing NK cell cytotoxicity, thereby increasing the production of cytokines such as tumor necrosis factor-α (TNF-α) and interferon-γ (IFN-γ) [25, 26]. Moreover, IL15 is not required for the maintenance of immune suppressive T cells, like T regulatory cells (Tregs), that can attenuate antitumor immune responses [26]. Additionally, the antitumor effect of IL15 has been well established in several mouse tumor models, with an IL15 deficiency possibly resulting in an acceleration of tumor growth [27–29]. In mice with CT26 colon cancer, IL15 inhibited tumor growth and prolonged the survival rate, thus emerging as a candidate for colon cancer treatment [30–32]. Despite this success, the systemic administration of IL-15 is known to cause considerable side effects, including weight loss, skin rash, hypotension, thrombocytopenia, liver injury, fever and rigors, and so on [30]; thus, a delivery method with reduced side effects is greatly needed.

The alpha isoform of the folate receptor (FRα) is associated with tumor cell proliferation, migration and invasion [33], with FRα overexpressed in approximately 30 – 40% of human colorectal carcinoma tissues (Supplementary Figure S1) [34, 35]. Elevated FRα expression in primary and metastatic colorectal carcinomas is significantly associated with a diminished 5-year disease-specific survival and premature patient death [36]. Therefore, FRα is a promising target for colon cancer-targeted therapy, with FRα-targeted non-viral vectors potentially having a place in colon cancer immunogene therapy. While a folate-modified micro-emulsifying drug delivery system for colon targeting has been tested *in vitro* [37], little has been reported regarding folate-modified lipoplexes for colon cancer immune gene therapy targeting *in vivo* [17].

In the present study, F-PLP/pIL15, a folate-modified lipoplex loading plasmid IL15 (pIL15) was constructed, and the physicochemical properties were characterized. Additionally, the antitumor effects and mechanisms of F-PLP/pIL15 were examined *in vivo* using a mouse CT26 colon cancer model that overexpresses FRα (Supplementary Figure S1).

## RESULTS

### Preparation and characterization of liposomes and lipoplexes

**Figure 1:**
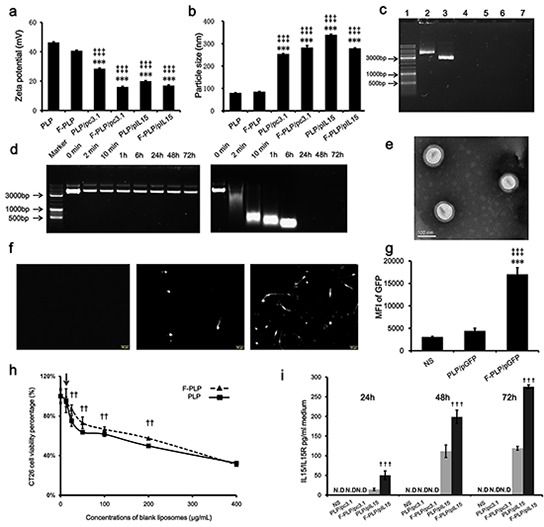
Physicochemical properties of F-PLP/pIL15 **a.** Zeta-potential of liposomes and lipoplexes (^***^P < 0.001, *versus* PLP; ^‡‡‡^*P* < 0.001, *versus* F-PLP; mean ± SD, n = 3). **b.** Particle size of liposomes and lipoplexes (^***^P < 0.001, *versus* PLP; ^‡‡‡^*P* < 0.001, *versus* F-PLP; mean ± SD, n = 3). **c.** Gel retardation assay of DNA and lipoplexes. Lane 1, DNA marker; lanes 2 and 3, naked pIL15 and pc3.1; lane 4, PLP/pIL15; lane 5, F-PLP/pIL15; lane 6, PLP/pc3.1; and lane 7, F-PLP/pc3.1. **d.** F-PLP/pIL15 stability in the presence of DNase. Left panel: F-PLP/pIL15 could protect pIL15 from DNase degradation for 72 h at 37°C; Right panel: naked pIL15 was completely degraded after a 2 min DNase incubation at 37°C. **e.** TEM image of F-PLP/pIL15. **f.** GFP expression fluorescent images (scale bars = 50 μm). **g.** Transfection efficiency of F-PLP/pDNA complexes in the CT26 cell line (MIF, mean fluorescence intensity; ^**^P < 0.01, *versus* NS; ^‡‡^*P* < 0.01, *versus* PLP/pGFP; mean ± SD, n = 3). **h.** IL15 expression detected by ELISA assay (N.D., not detected; ^†††^*P*<0.001, *versus* PLP/pIL15; mean ± SD, n = 3). **i.** The cytotoxicity of liposomal carriers on CT26 cells (arrow represents 12.5μg/mL, which was the concentration of liposomal carriers used in transfection test *in vitro*; ^††^*P* < 0.01, *versus* PLP; mean ± SD, n = 3).

PLP and F-PLP were produced using a film hydration method as previously described [17, 19, 38]. The Zeta potential values of the blank liposomes (Figure 1a), both PLP and F-PLP, were higher than that of the pDNA-liposome complexes (F-PLP/pIL15, F-PLP/pc3.1, PLP/pIL15 and PLP/pc3.1), which had a lipid/DNA mass ratio of 6:1. This indicates that when negatively charged plasmid DNA bound with a cationic liposome, the positive charge of the liposome was partially neutralized, thus resulting in a decreased positive charge.

When examining the sizes of blank liposomes, they were both found to be about 80 – 85 nm (Figure 1b), while the DNA-liposome complexes were significantly larger reaching 250 – 300 nm (*P* < 0.001). DNA-liposome complexes are formed by electrostatic interaction between DNA and two or more cationic liposomes. These findings suggest that these complexes may contain two or more cationic liposomes, thereby exhibiting a significant increase in size relative to the blank liposome. Additionally, the polydispersion indexes (PDIs) of the blank liposomes (F-PLP and PLP) and lipoplexes (F-PLP/pIL15, and P-LP/ pIL15) were about 0.2 – 0.3. Transmission electron microscopy (TEM) was used to analyze the morphology of the F-PLP/pIL15 lipoplex and showed a spheroidal shape and uniform-size (Figure 1e). Agarose gel electrophoresis, with a nanogram grade sensitivity (Supplementary Figure S2), was used to characterize the encapsulation efficiencies of the liposome-DNA complexes. As expected, DNA bound to two or more cationic liposomes was unaffected by the electric field and unable to be stained effectively; thus, the bound DNA were not visible on the gel [39]. Within the obtained gel image (Figure 1c), the bright band was defined as free DNA (lanes 2 and 3) and almost no bright bands appeared in lanes 4, 5, 6, and 7. The lipid/DNA mass ratio was 6:1, and the total free pDNA in the mixture was under the detection limit (< 7.815ng). Thus, these results confirmed that the DNA was mostly bound with the liposomes.

The stability of the pDNA in the F-PLP/IL15 complex was assessed in the presence of DNase I (Figure 1d). Naked pIL15 was completely degraded after 2 min of incubation with DNase I, while the F-PLP/IL15 complex prevented pIL15 degradation for 72 h at 37°C, thus increasing stability. When examining expression levels, F-PLP remarkably increased the reporter gene expression (Figure 1f and 1g; *P* < 0.01) relative to PLP. The mean fluorescence intensity (MIF) of GFP was determined by FACS flow cytometry and was three times higher in F-PLP/GFP (MIF = 17031) treated wells compared with the PLP/GFP (MIF = 4440) treatment. The percentage of GFP-positive cells after transfection by F-PLP/GFP was 12.1%, almost twice that of those transfected by PLP/GFP (data not shown). These findings were further supported by observations under the fluorescence microscope (Figure 1f).

Cytotoxicity was assessed after treatment with different liposomes for 48 h (Figure 1h) and showed that the CT26 cell viability percentages were dependent on the liposome concentration. At the lowest concentration (12.5 μg/mL; the concentration used in transfection test *in vitro*), the cytotoxicity of PLP was similar to that of F-PLP. When the cells were treated with higher concentration of liposomes (25 – 200 μg/mL), F-PLP liposomes with a lower Zeta potential showed a lower toxicity than PLP liposomes (*P* < 0.01). These findings indicate that F-PLP may be a suitable low-toxicity gene carrier for use in colon cancer therapy. The IL15 expressed by colon cancer cells is only secreted in a complex with its receptor (IL15/IL15R); thus, an ELISA kit measuring IL15/IL15R was used to determine IL15 expression levels. This assay showed that F-PLP significantly enhanced IL15/IL15R secretion *in vitro* compared to PLP (Figure 1i; *P* < 0.001), with IL15 secretion present in a time dependent manner. While these findings show that F-PLP can transfer pIL15 to colon cancer cells effectively, the antitumor effect of F-PLP/pIL15 requires further study using a murine model.

### *In vivo* antitumor effect and IL15 secretion induced by F-PLP/pIL15

**Figure 2:**
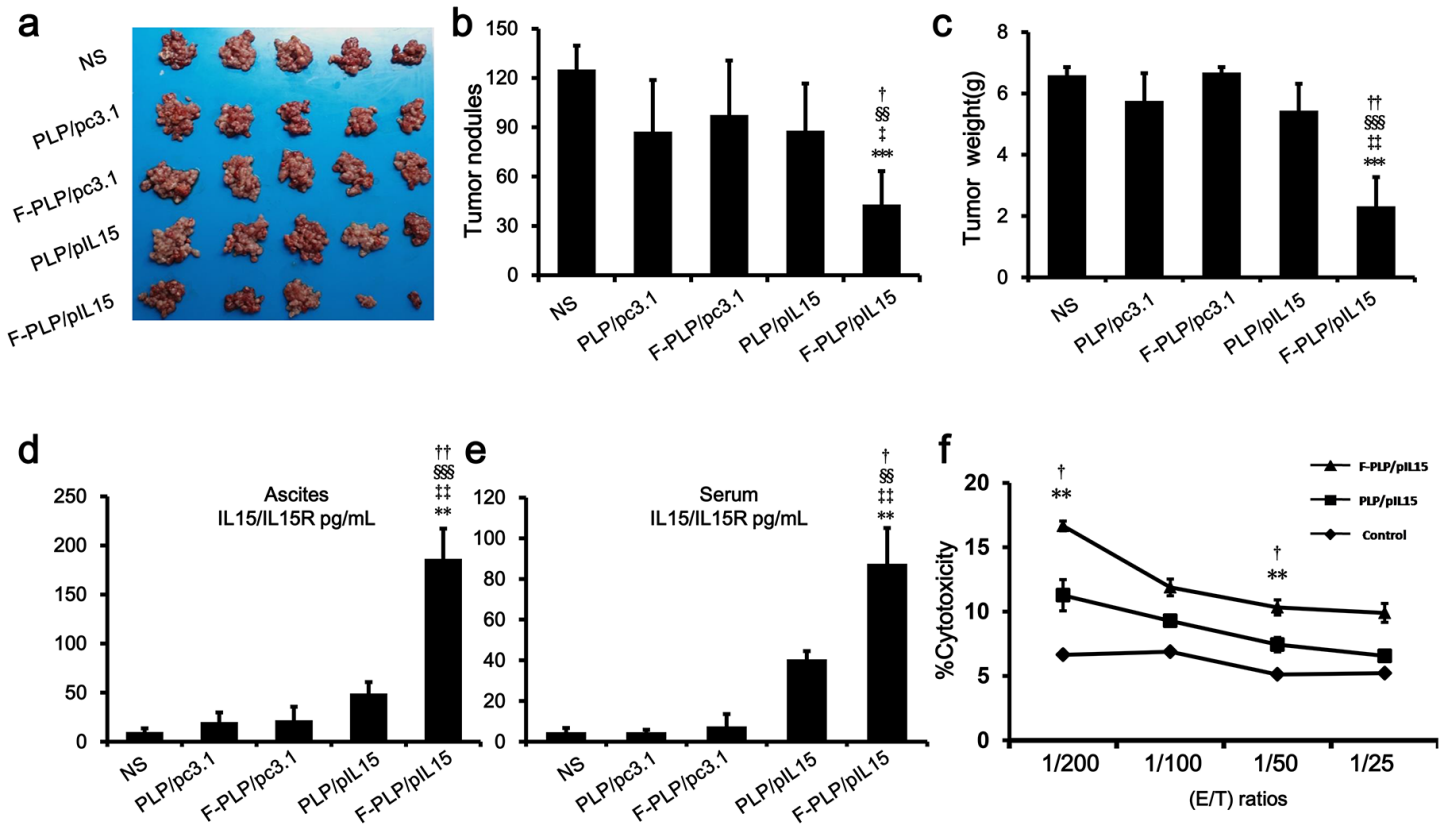
*In vivo* antitumor effect of F-PLP/pIL15 and pIL15 expression **a.** Tumor images; **b.** tumor nodules; and **c.** tumor weight analysis. **d.** Serum and **e.** ascite IL15 expression in tumor-bearing mice. **f.** The spleen cell-mediated cytotoxicity evaluation by a ^51^Cr release assay. As a result, the spleen cell-mediated cytotoxicity was in concordance with the expression of IL15. (^**^P < 0.01, ^***^P < 0.001, *versus* NS; ^‡^*P* < 0.05, ^‡‡^*P* < 0.01, ^‡‡‡^*P* < 0.001, *versus* PLP/pc3.1; **^§§^***P* < 0.01, **^§§§^***P* < 0.001, *versus* F-PLP/pc3.1; ^†^*P* < 0.05, ^††^*P* < 0.01, *versus* PLP/pIL15; mean ± SD, n = 3 or 5).

Compared with PLP/pIL15, F-PLP/pIL15 was capable of remarkably inhibiting tumor growth by reducing the number of tumor nodules from 88 to 43 (Figure 2a and 2b; *P* < 0.05) and tumor weight from 5.43 g to 1.88 g (Figure 2a and 2c; *P* < 0.01). Moreover, when examining the inhibition of malignant ascites, the F-PLP/pIL15 group was found to have a smaller ascites volume than the other three lipoplex groups (PLP/pc3.1, F-PLP/pc3.1 and PLP/pIL15; data not shown). Furthermore, the two vector treatment groups, PLP/pc3.1 and F-PLP/pc3.1, generated a weak antitumor effect compared to the NS group (Figure 2a – 2c), which may be due to nonspecific cytotoxicity in liposomes or lipoplexes containing DOTAP [40].

The antitumor effect induced by F-PLP/pIL15 could be attributed to the higher IL15 expression and its subsequent immune actions. Therefore, pIL15 expression was examined *in vivo* in a murine model. On the 24th day after the i.p. administration with PLP/pIL15 or F-PLP/pIL15, IL15 expression in ascites and the serum of tumor bearing mice was determined by ELISA analysis. After receiving 8 doses, the mean IL15/IL15R concentrations in F-PLP/pIL15-treated mice were 186 pg/mL in ascites and 87 pg/mL in the serum, which was significantly higher than the levels seen in PLP/pIL15-treated mice (Figure 2d and 2e; 49 pg/mL,^††^*P* < 0.01 and 40 pg/mL, ^†^*P* < 0.05, respectively), These results revealed that i.p. administration of F-PLP/pIL15 generated higher IL15 expression levels in both ascites and the serum. This increased localized expression of IL-15 in the tumor microenvironment (ascites) could enhance antitumoral T-cell responses, while the increased serum levels may aid in the prevention of metastasis, thus jointly inhibiting tumor growth [28].

The differences in IL15 expression induced by these two lipoplexes is most likely attributed to specific interaction mediated by the additional folate-moiety in F-PLP/pIL15. Additionally, the overexpresssion of FRα on the tumor cell surface may also contribute to a more effective uptake and expression of pIL15. All these results indicated that F-PLP/pIL15 is an effective gene carrier able to target colon cancer for immune gene therapy.

### Influence of IL15 on spleen immune cells

**Figure 3:**
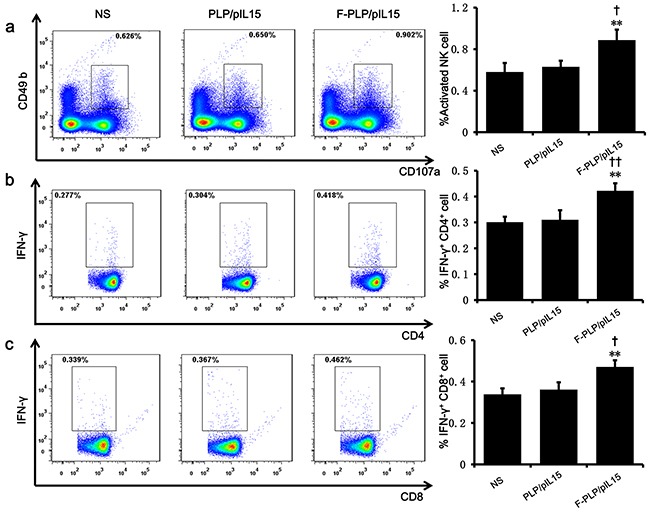
Induction and activation of NK cells and the increasing of IFN-γ^+^ cytotoxic T cells **a.** A splenic single cell suspension was analyzed by flow cytometry for the presence of activated NK cells CD49b^+^CD107a^+^. The increase of CD4^+^IFN-γ^+^ **b.** and CD8^+^IFN-γ^+^ **c.** cytotoxic T cells were examined with quantitative flow cytometry (^**^P < 0.01, ^***^P < 0.001, *versus* NS; ^†^*P* < 0.05, ^††^P < 0.01, *versus* PLP/pIL15; mean ± SD, n=3).

Herein we hypothesized that antitumor effect induced by IL15 was due to NK and T cell activation [41, 42]. Since the spleen contains many immune cells, including NK cells and T cells, spleen cell-mediated cytotoxicity was examined via a ^51^Cr release assay to determine levels of NK and T cell activation. This assay showed that F-PLP/pIL15 increased spleen cell-mediated cytotoxicity (Figure 2f). At effector to target (E/T) ratios of 200/1 or 50/1, the splenocytes from F-PLP/IL15 treated mice shown an elevated cytotoxic activity relative to PLP/IL15 treated mice (*P* < 0.05). In agreement with the cytotoxicity assay results, a higher ratio of activated NK cells to total spleen cells was seen in the F-PLP/pIL15 group (0.886%) than in the PLP/pIL15 group (0.629%, ^††^P < 0.01; Figure 3a). However, the ratio of the total number of NK cells to spleen cell was not significantly different between the PLP/pIL15 group (4.84%) and F-PLP/pIL15 group (5.01%, *P* = 0.503; Supplementary Figure S3). Therefore, these results suggest that the increased spleen cell-mediated cytotoxicity may be attributed to an increased activation rather than an increased proliferation.

When examining spleen IFN-γ^+^cytotoxic T cells of subtype CD4^+^ and CD8^+^, F-PLP/pIL15-treated mice showed increased CD4^+^ T cells levels (0.422%) and CD8^+^ T cells levels (0.470%) relative to the PLP/pIL15-treated mice (0.310% and 0.361%, *P* < 0.01; Figure 3b and 3c). Thus, it would appear that the increased NK cell activation and IFN-γ^+^ cytotoxic T cells were induced by the secretion of IL15 in F-PLP/pIL15-treated tumor cells, thus generating an antitumor effect.

### Induction of cellular apoptosis and the inhibition of cellular proliferation

**Figure 4:**
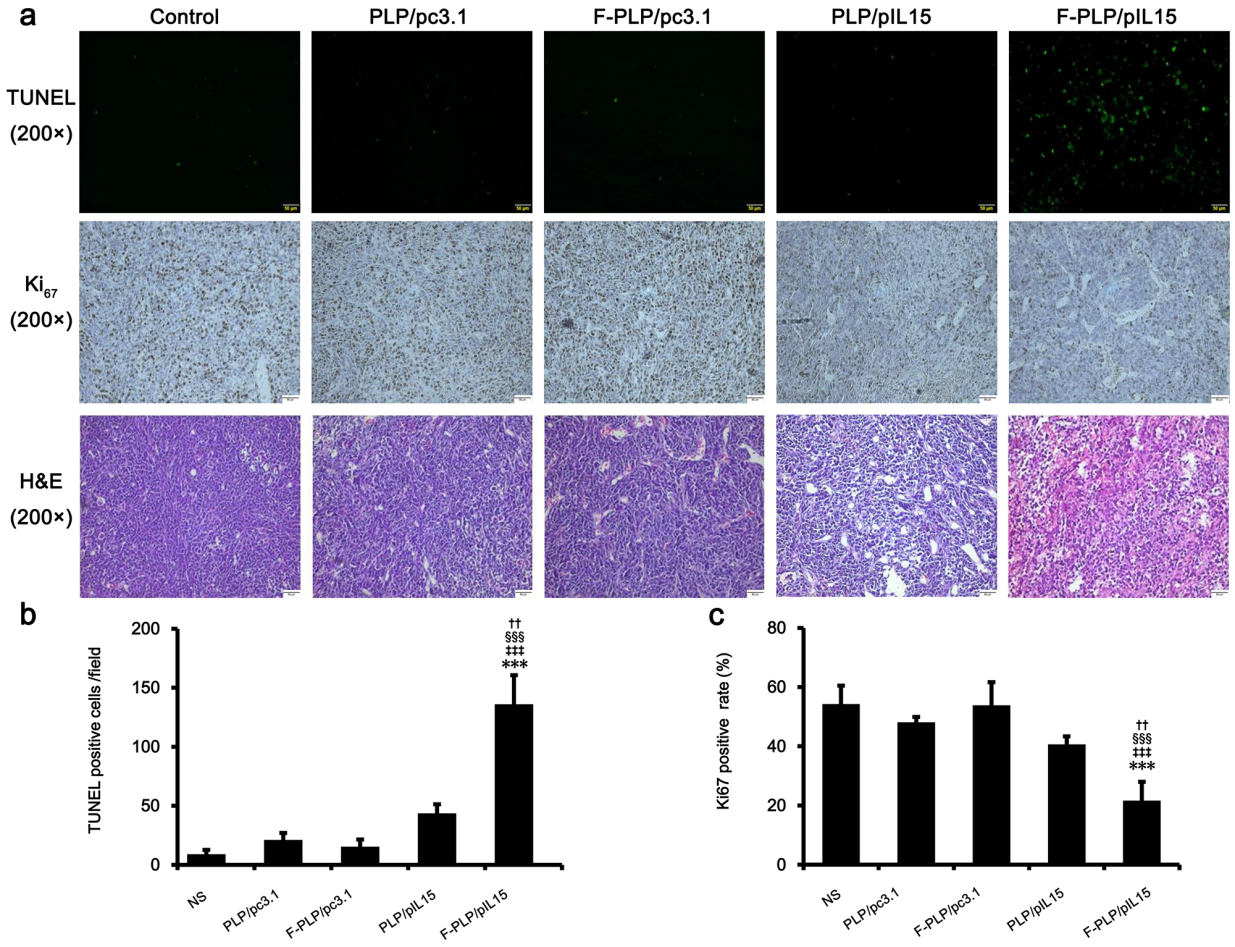
Antitumor mechanisms of F-PLP/pIL15 **a.** Representative tumor tissue sections following the TUNEL assay, Ki_67_ staining and hematoxylin-eosin (H&E; scale bars = 50 μm). **b.** and **c.** Tumor cell apoptosis and proliferation were assessed by counting the number of TUNEL-positive cells per field and the Ki_67_-positive index rate (three high power fields per slide). F-PLP/pIL15 was superior to the controls in increasing tumor apoptosis and inhibiting tumor cell proliferation (^*^P < 0.05, ^**^P < 0.01, *versus* NS;^‡^*P* < 0.05, ^‡‡^*P* < 0.01, *versus* PLP/pc3.1; ^§^*P* < 0.05, ^§§^*P* < 0.01, *versus* F-PLP/pc3.1; ^†^*P* < 0.05, *versus* PLP/pIL15; mean±SD, n = 3).

Tumor growth is considered a destruction of the balance between apoptosis and proliferation. To explore whether some phenotypical changes occurred in the tumor tissues, the percentage of apoptotic and proliferating cells were examined. To determine apoptotic levels, a TUNEL assay was employed. This assay aids in the detection of early DNA fragmentation associated with apoptosis and was employed to determine if this mechanism was associated with the antitumor effects of F-PLP/pIL15 treatment observed *in vivo*. In the F-PLP/pIL15-treated group, 136 TUNEL-positive cells/field were identified, which was a 3-fold increase from levels seen in the PLP/pIL15-treated group (44 TUNEL-positive cells/field, *P* < 0.05; Figure 4a and 4b). This significant increase suggests that F-PLP/pIL15 might offer an additional antitumor effect via interactions between folate and FRα.

To assess tumor cell proliferation levels, Ki_67_ staining was performed. These results showed fewer proliferating cells (brown) in tumor tissues treated with F-PLP/pIL15 (16%) than in those treated with PLP/pIL15 (37%, *P* < 0.05; Figure 4a and 4c) or in the control group (65%, *P* < 0.01). Moreover, higher levels of necrotic tumor cells were observed in F-PLP/pIL15-treated tumor tissues following H & E staining (Figure 4a). Combined, this evidence suggests that F-PLP/pIL15 can successfully deliver pIL15 to tumor cells via folate and FRα interactions and generate antitumor effects by activating immune cells, inducing tumor cytotoxicity and cellular apoptosis and inhibiting tumor cell proliferation.

### F-PLP/pIL15 safety and toxicity assessment in mice

**Figure 5:**
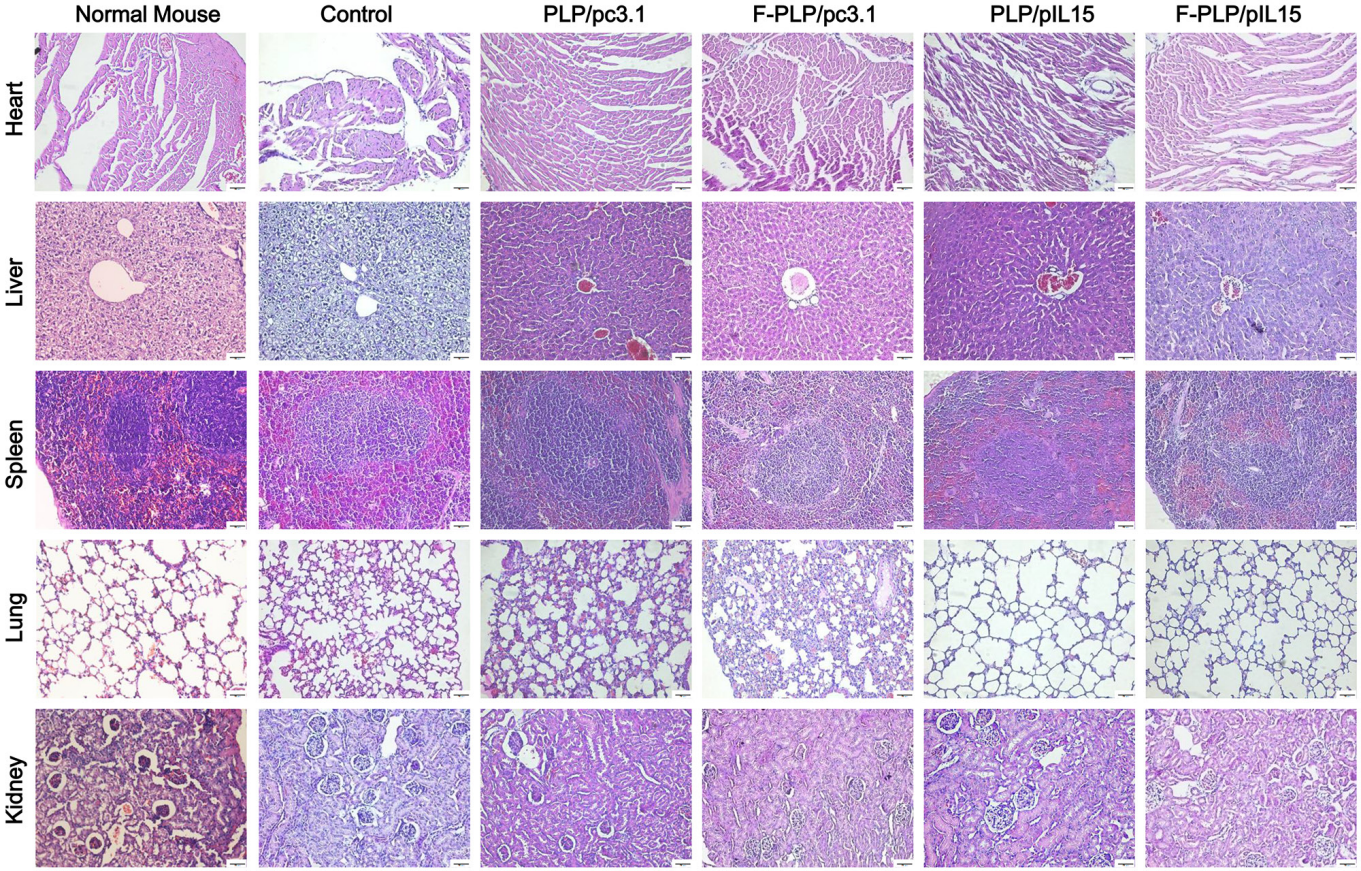
Histological examinations of H&E-stained vital organ sections Following CT26 tumor inoculation, an inflammatory response was observed in the lungs when compared to normal BALB/c mice. This inflammatory response was reduced following gene therapy. No significant pathological differences were observed in the five groups (scale bars = 50 μm).

To examine potential lipoplex toxicity, some key mice organs, to include the heart, liver, spleen, lung and kidney, were harvested and H & E stained for histopathological analysis, with no significant pathological changes noted (Figure 5). Additionally, no obvious toxicities were observed in the mice, as determined by appearance, fecal examination and urinary excretion.

## DISCUSSION

In the current study, a folate-modified liposome was used as an IL15 gene carrier. Compared with non-targeted liposome (PLP), the targeted liposome (F-PLP) not only increased the transfection efficacy of the reporter gene, but also enhanced the expression and secretion of the IL15 gene in colon cancer cells *in vitro*. A CT26 murine colon cancer model was used to evaluate the potency of F-PLP/pIL15. *In vivo* antitumor experiments showed that F-PLP/pIL15 could inhibit tumor growth, reduce the number of tumor nodules and reduce the tumor weight. Compared with PLP/pIL15, F-PLP/pIL15 also increased the IL15 levels in both the ascites and the serum. Collectively, it would appear that all of these antitumor effects may be attributed to the successful delivery of pIL15 to tumor cells via interactions between F-PLP/pIL15 and FRα.

Furthermore, the increased IL15 secretion in F-PLP/pIL15-treated mice and suspected subsequent immune reactions, specifically NK cell activation and increased cytotoxic T cells, were confirmed following a spleen cell-mediated cytotoxicity assayed monitoring ^51^Cr release. Furthermore, this observed increase in activated NK cells and cytotoxic T cells was further confirmed by flow cytometry. These results suggested that the F-PLP/pIL15 complex can successfully induce NK cell activation and increase cytotoxic T cells, thus generating an antitumor effect. Furthermore, the examination of any potentially adverse side effects following F-PLP/pIL15 treatment in mice showed no obvious toxicity. In conclusion, this study suggests that F-PLP/pIL15 is an efficient and safe formulation when i.p. administered and may serve as a potential targeting preparation for colon cancer immunotherapy.

## MATERIALS AND METHODS

### Materials

1,2-Dioleoyl-3-trimethylammoniumpropane (DOTAP) was purchased from Lipoid GmbH (Ludwigshafen, Germany). Cholesterol (Chol) was obtained from Shanghai Bio Life Science and Technology Co. Ltd. (Shanghai, China). Methoxy poly (ethylene glycol)-succinyl-cholesterol conjugate (mPEG-suc-Chol) and folate-poly (ethylene glycol)-succinyl-cholesterol conjugate (F-PEG-suc-Chol) were synthesized as previously described [19, 43, 44]. A DNA ladder, Gene Ruler DNA ladder mix (SM0331) and loading buffer were obtained from Fermentas (Thermo Fisher Scientific Inc., Waltham, MA, US). 3-(4,5-Dimethyl-2-thiazolyl)-2,5-diphenyl- 2H-tetrazolium bromide (MTT) were purchased from Sigma-Aldrich, Co. (St. Louis, MO, US). DNase I was purchased from Roche diagnostics GmbH (Roche Applied Science Mannheim, Germany). Triton X-100 was obtained from Sanland Chemical Co., Ltd. (Los Angeles, CA, US). Anti-FOLR1 antibody (LSB5727) was provided from LifeSpan Biosciences (Seattle, WA, US). All the reagents used in this study were analytical grade (AR).

### Murine colon cancer model establishment

Murine IL15 (pIL15) was cloned into the pcDNA3.1 (pc3.1) expression vector (Invitrogen Corp., Carlsbad, CA, US) [45]. All plasmids used in this study were purified with an Endo-free Giga kit (Qiagen, Hilden, Germany) according to the manufacturer's instructions.

CT26 colon carcinoma cells and YAC-1 lymphoma cells were obtained from American Type Culture Collection (Manassas, VA, US), cultured in RPMI-1640 medium supplemented with 10% fetal bovine serum (FBS; Huhhot Caoyuan lvye Bio-engineering material Co., Ltd., Huhhot City, Inner Mongolia Autonomous Region, China) and maintained at 37°C with 5% CO_2_.

All animal experiments were performed in accordance with the guidelines of Sichuan University and approved by the Animal Care Committee of Sichuan University (Chengdu, China). Female BALB/c mice (6 – 8 weeks old) were purchased from Vital River (Beijing, China) and housed in a specific-pathogen-free (SPF) environment, with a consistent room temperature and humidity, and handled in strict accordance with good animal practice. *In vivo* colon cancer mouse models were established by intraperitoneal (i.p.) injection of CT26 cells (about 2 × 10^5^ cells/0.2 mL serum-free RPMI-1640), with mice then randomly divided into five groups (NS, PLP/pc3.1, F-PLP/pc3.1, PLP/pIL15, and F-PLP/pIL15).

### Preparation and characterization of liposomes and lipoplexes

Folate-modified PEGylation liposome (F-PLP) and non-folate-modified PEGylation liposome (PLP) were prepared using a film dispersion method as previously described [17, 46, 47]. In brief, DOTAP, Chol, mPEG-suc-Chol and F-PEG-suc-Chol were dissolved in chloroform and the solution was evaporated on a rotary evaporator to remove the organic solvent. The thin film that was formed was further dried under high vacuum for 6 h and hydrated in 5% (weight in volume) glucose solution. The lipid suspension was then probe sonicated until a translucent lipid suspension was observed, sterilized via filtration through a 0.22 μm microporous membrane (Millipore Ireland BV, Carrigtwohill, Co. Cork, Ireland) and stored at 4°C until further use.

Lipoplexes were formed by combining the obtained liposomes with plasmid for 30 min at room temperature. Prior to combination, the plasmid DNA was pre-melted via ice bath and 2 μL (1 μg/μL) plasmid DNA was diluted with 48 μL 5% glucose solution, while 12 μL (1 μg/μL) liposome was diluted with 38 μL 5% glucose solution. Both dilutions were gently mixed by pipetting 3 – 5 times, incubated at room temperature for 30 min and the obtained lipoplex was used to evaluate physicochemical properties, *in vitro* cell transfection or *in vivo* gene therapy. The mean particle size and Zeta potential of the liposomes and lipoplexes were measured via Zetasizer Nano ZS ZEN 3600 (Malvern Instruments, Ltd., Malvern, Worcestershire, U.K.). Electrophoresis was performed on a 0.8% (w/v) agarose gel (Invitrogen Corp, Carlsbad, CA, US) in pH 7.4 TAE buffer (40mM Tris/HCl, 1% acetic acid, 1 mM EDTA) with GoldView^TM^ used as a nucleic acid stain. The gels were visualized and digitally imaged using a gel documentation system (Gel Doc 1000, Bio-Rad Laboratories, Hercules, CA, US).

To characterize complex stability, DNase degradation was carried out as previously described [43]. Briefly, F-PLP/pIL15 containing 20 μg pIL15 or 20 μg naked pIL15 DNA were incubated with 1U of DNase I in a total volume of 400 μL in a 50 mM Tris buffer (pH 7.4) containing 10 mM MgCl_2_. This mixture was incubated at 37°C and aliquots of 40 μL were taken at 2 min, 10 min, 1 h, 6 h, 24 h, 48 h and 72 h, with 4 μL of 250 mM EDTA added immediately to stop degradation and all samples placed in an ice bath. To cause the dissociation of pIL15 from the F-PLP/pIL15 complex, 2 μL of Triton X-100 was added and allowed to incubate for 5 min at room temperature. Next, 4 μL of 1 mg/mL sodium heparin was added and allowed to incubate at room temperature for 15 min. Lastly, 10 μL of each sample was utilized for gel electrophoresis as described above.

Lipoplex morphology was observed using transmission electron microscopy (TEM; FEI Tecnai G 2 F20, Hillsboro, OR, US). The samples were diluted with distilled water, placed on a copper grid and negatively stained with molybdophosphoric acid for 1 min. The grid was then allowed to dry at room temperature and examined using the TEM.

### *In vitro* gene transfer and expression

CT26 cells were seeded on a Costar 6-well plate (Corning Incorporated, Corning, NY, USA) at a density of 1.5×10^5^ cells/well in 2 mL of complete RPMI-1640 culturing medium. After a 24 h incubation, the medium was replaced with 800 μL folate- and serum-free RPMI-1640 medium per well. F-PLP/pGFP, PLP/pGFP, F-PLP/pc3.1, PLP/pc3.1, F-PLP/pIL15 or PLP/pIL15 in a final volume of 200 μL and containing 2 μg plasmid DNA were subsequently added to designated wells and allowed to incubate for 6 h. Post-incubation, the medium was returned to complete culture medium. GFP transfected cells were observed under a fluorescent microscope (Olympus IX73, U-HPLGPS, Olympus Corporation, Shinjuku, Tokyo, Japan) following an additional 42 h incubation and the transfection efficiency was determined via FACS flow cytometry (BD Biosciences, San Jose, CA, USA). In cells transfected with F-PLP/pc3.1, PLP/pc3.1, F-PLP/pIL15 or PLP/pIL15 for 24, 48 or 72 h, IL-15 expression levels were determined using a mIL-15 ELISA kit (eBioscience Inc., San Diego, CA, U.S.) according to the manufacturer's instructions, with normal saline (NS) used as a negative control.

### PLP and F-PLP cytotoxicity assay via MTT

PLP and F-PLP cytotoxicity was evaluated in the CT26 cell line by MTT assay as previously described [48]. Briefly, cells were seeded on 96-well plates (Corning Inc., NY, US) at a density of 3,000 cells/ well with 100 μL RPMI-1640. Following attachment overnight, cells were treated with another 100 μL of various concentrations of PLP or F-PLP diluted in RPMI-1640 and the mixture was further incubated for another 48 h to assess concentration dependent and time-dependent cytotoxicity. After the cells were cultured for a predetermined time, 20 μL of MTT stock solution (5 mg/mL in saline) was added to each well and the cells were further cultured at 37°C for an additional 4 h. Finally, the culture medium was removed by aspiration and 150 μL of DMSO was added to each well to dissolve the formazan crystals.

The absorbance of each well was read at 570 nm on a Multiskan MK3 microplate reader (Thermo Fisher Scientific Inc., Waltham, MA, US). In this assay, absorbance was proportional to number of viable cells. Untreated cells were used as a control and a relative cell viability was determined relative to the control as follows:Relative cell viability (%) = *A*_treated_/*A*_control_ × 100

### Treatment with various liposome-pDNA complexes in a murine colon cancer model

Liposome-pDNA complexes were prepared as above and administered intraperitoneally three days after tumor cell injection. The liposome-pDNA complexes containing 10 μg plasmid in a 200 μL volume were given every two days for a total of 8 doses. Mice were monitored daily for adverse therapeutic effects. At the time of sacrifice (48 h after the final dose), tumor tissues, the spleen and other vital organs were harvested and the total mouse weight, ascetic fluid volume and tumor weight, were recorded. The tumor nodules were separated from the peritoneal cavity, cleared with 0.9% NaCl solution and counted by two researchers using a double blind method. Finally, a third researcher calculated and recorded the number of tumor nodules in each group. The antitumor effect was determined by the tumor nodule numbers and weight and by the ascetic fluid volume.

### Flow cytometry analysis and spleen cell-mediated cytotoxicity assay

Mice spleens from the control, PLP/pc3.1, F-PLP/pc3.1, PLP/pIL15 and F-PLP/pIL15 were harvested and the spleens were ground with a pestle and filtrated with cell strainer (BD Biosciences, San Jose, CA, US) to form a splenic single cell suspension. A subset of these cells were harvested and stained with CD107a-PE (BD Biosciences 10 μL/mL) in the presence of interleukin-2 (100 U/mL) and monensin (Golgi-Stop, BD Biosciences) for 4 h at 37°C in 5% CO_2_.

CD107a (lysosome-associated membrane protein-1, LAMP-1) is transported to the surface of the NK cells during activation, making it an attractive surface marker for measuring NK cell activity [49, 50]. Phorbol-12-myristate-13-acetate (PMA, 2.5 μg/mL; Sigma Chem. Co., St. Louis, MO, US) and ionophore (Ionomycin, 0.5 μg/mL; Sigma Chem. Co., St. Louis, MO, US) were used as positive controls. After the culture, cells were then stained with CD49b-FITC (BD Biosciences) and CD3-Percp (BD Biosciences) for 30 min at 4°C. NK cells were determined as CD3^−^CD49b^+^ and active NK cells were determined as CD49b^+^CD107a^+^. Another subset of the spleen cells were stained for T cell marker CD4-APC (BD Biosciences) and CD8-FITC (BD Biosciences). The expression of IFN-r was determined by IFN-r-PE (BD Biosciences) staining for 30 min at 4°C after samples were fixed/ permeabilized with paraformaldehyde and Triton-X 100.

Spleen cells were added to the YAC-1 target cells, which were pre-incubated with ^51^Cr at effector to target (E:T) ratios of 200:1, 100:1, 50:1, and 25:1. After 4 h, splenocyte cytotoxicity was measured via liquid scintillation counter, with 100 μL of supernatant containing 5 × 10^3^ target cells per well. The cytotoxic activity was then calculated by the following formula:

$$\%\text{ Cytotoxicity}=\frac{\text{Experimental release}-\text{spontaneous release}}{\text{Maximum release}-\text{spontaneous release}}\times 100\%$$

### Proliferation and apoptosis assays of tumor tissues

Tumor tissue proliferation was immunohistochemically analyzed using a rabbit anti-human Ki_67_ antibody (Novus Biologicals, Littleton, CO, US) with a streptavidin-biotin detection method. Ki_67_ expression was quantified by counting the number of positive cells in 10 randomly selected fields at a 200× magnification. Tumor apoptotic levels were determined using a terminal deoxynucleotidyl transferase-mediated nick end labeling (TUNEL) immunofluorescence kit (Promega, Madison, WI, US) according to the manufacturer's instructions. TUNEL-positive cells were defined as cells with pyknotic nuclei, thus dark green fluorescent staining. Apoptotic cells were counted under an Olympus BX53 microscope with a mercury lamp (U-HGLGPS; Olympus Corporation, Shinjuku, Tokyo, Japan) at a 200x magnification in randomly selected fields.

### Toxicity assessment

To evaluate potential treatment associated side effects and toxicity, relevant indices such as weight loss, diarrhea, anorexia, cachexia, skin ulcerations and toxic deaths were monitored. Heart, liver, spleen, lung and kidney tissues were harvested, fixed in 4 % paraformaldehyde solution, embedded in paraffin and sectioned at 5 μm. The sections were then H & E stained and observed by two pathologists in a blinded manner.

### Statistical analysis

Statistical analysis was performed using a one-way ANOVA in Statistical Product and Service Solutions software (SPSS, V 19.0; IBM Corp., New York, US). When equal variances were assumed after a homogeneity of variance test, the LSD multiple comparisons test was used. When equal variances were not assumed after a homogeneity of variance test, a Dunnett T3 multiple comparisons test was used. Differences were considered statistically significant at *P* < 0.05.

## SUPPLEMENTARY FIGURES


