# Supplementary material for: A folate receptor-targeted lipoplex delivering interleukin-15 gene for colon cancer immunotherapy
Source: Oncotarget. 2016 Jul 11;7(32):52207–17. doi: 10.18632/oncotarget.10537 (PMC5239545; doi:10.18632/oncotarget.10537)
Supplement: Supplementary file 1 [file oncotarget-07-52207-s001.pdf]

A folate receptor-targeted lipoplex delivering interleukin-15 gene for colon cancer immunotherapy

SUPPLEMENTARY FIGURES

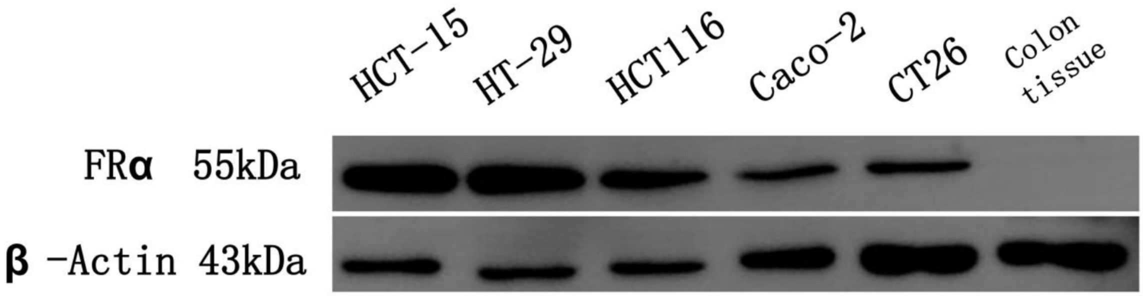

Supplementary Figure S1: Expression of folate receptor α (FRα) in colon cancer cell lines and normal tissue. HCT-15, HT-29, HCT 116 and Caco-2 cell lines from human colorectal adenocarcinoma, CT26 cell line from BALB/c mouse colon carcinoma, and colon tissue from BALB/c mouse. Anti-FRα antibody (LSB5727) was provided from LifeSpan Biosciences (Seattle, WA, US). β-Actin (sc-69879) was purchased from Santa Cruz Biotechnology, Inc. (Dallas, TX, US).

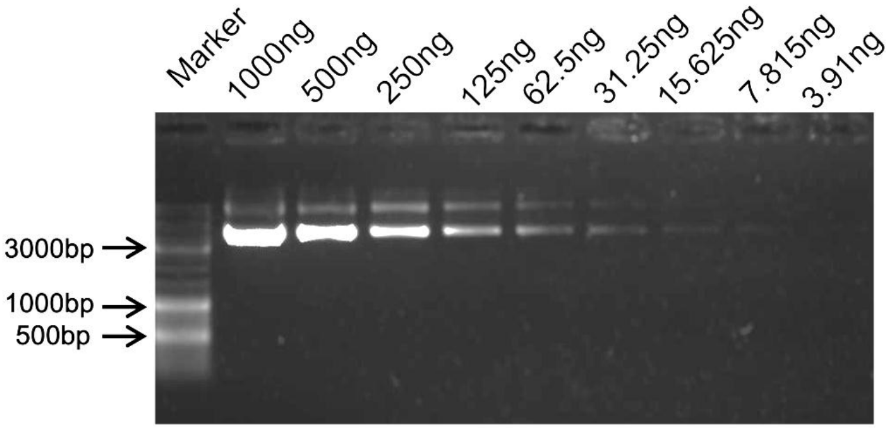

Supplementary Figure S2: The sensitivity test of agarose gel electrophoresis. The agarose gel electrophoresis method used in this study is sensitive enough to detect the DNA in nanogram grade (<7.815ng).

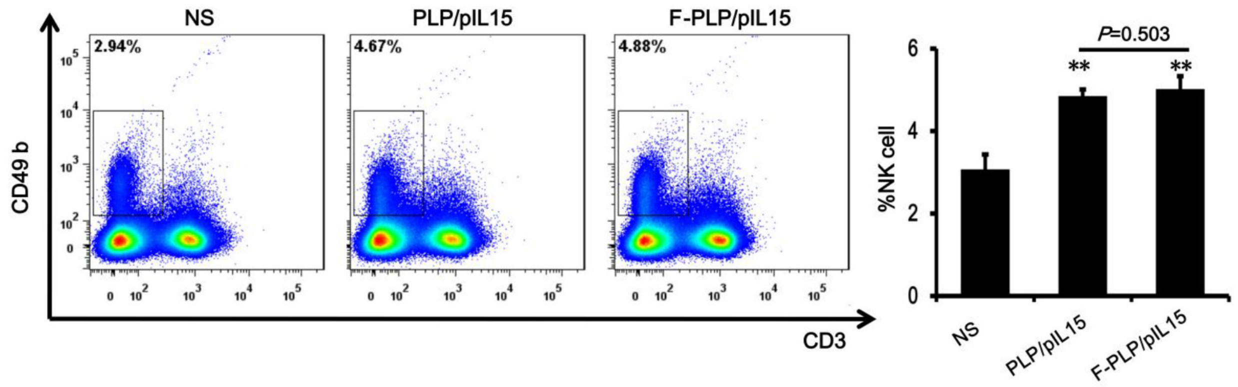

Supplementary Figure S3: NK proliferation mediated by lipoplexes. Compared with that in NS mice (3.07%), the rates of NK cells in both PLP/pIL15(4.84%) and F-PLP/pIL15 (5.01%)-treated mice were remarkably raised (***P* < 0.01, *versus* NS, Mean ± SD, n = 3). However, F-PLP/pIL15 treatment didn't show significant advantage over PLP/pIL15 treatment (*P*=0.503, Mean ± SD, n = 3).
